# Proteome-Scale Discovery and Multilevel Validation of Antioxidant Short Peptides from *Apostichopus japonicus*: Keap1 Interactions and Cellular Antioxidant Responses

**DOI:** 10.3390/antiox15091188

**Published:** 2026-09-17

**Authors:** Jingxian Zheng, Yijia Tang, Shu Xing, Weiwei Han

**Affiliations:** Key Laboratory for Molecular Enzymology and Engineering of Ministry of Education, School of Life Sciences, Jilin University, Changchun 130012, China; jxzheng24@mails.jlu.edu.cn (J.Z.); tangyj1323@mails.jlu.edu.cn (Y.T.)

**Keywords:** *Apostichopus japonicus*, antioxidant peptides, in silico screening, Keap1/Nrf2, molecular dynamics

## Abstract

Antioxidant peptides from marine proteins are promising bioactive compounds, yet efficient discovery and multilevel validation remain challenging. Here, we established a proteome-scale workflow integrating virtual enzymatic hydrolysis, dual-model antioxidant prediction, consensus ranking, toxicity screening, sequence-diversity-informed selection, experimental validation, and molecular simulation to identify antioxidant short peptides from *Apostichopus japonicus*. Five newly identified antioxidant short peptides (DHE, HDHE, DDHN, EHED, and QHDE) were synthesized for experimental validation. All showed DPPH and ABTS radical-scavenging activity, good cytocompatibility, and protection against D-galactose-induced loss of HepG2 cell viability. HDHE showed the highest DPPH activity and QHDE showed the highest ABTS activity at higher concentrations, whereas EHED produced the greatest recovery of cell viability. Molecular docking and 100 ns molecular dynamics simulations indicated sequence-dependent Keap1 interaction patterns and preserved global receptor stability, but these computational features did not parallel cytoprotection. Western blotting further showed differential regulation of Keap1, Nrf2, and HO-1, with DDHN, EHED, and QHDE significantly increasing HO-1 expression. Across the workflow, predicted activity, chemical radical scavenging, Keap1 interaction characteristics, and cellular protection did not converge on the same peptide. These findings establish a proteome-scale discovery strategy and show that multilevel validation is essential because computational, chemical, molecular-interaction, and cellular readouts represent complementary rather than interchangeable dimensions of antioxidant peptide performance.

## 1. Introduction

Food-derived antioxidant peptides can influence redox homeostasis through both chemical reactivity and cellular responses. Reactive oxygen species (ROS) can damage proteins, lipids, and nucleic acids when their production exceeds endogenous antioxidant capacity, whereas antioxidant defenses continuously limit these effects. Peptides can contribute through radical scavenging, electron or hydrogen donation, metal-ion coordination, and interactions with cellular proteins involved in redox regulation [1,2]. Their compact sequences also allow multiple chemically distinct residues to contribute within the same molecule. Food-derived peptides have been shown to alleviate oxidative injury in HepG2 and other cellular models, indicating that their biological effects extend beyond cell-free radical-scavenging assays [3,4,5]. However, chemical antioxidant capacity does not necessarily predict cellular protection because peptide stability, sequence context, accessibility to radicals, and target interactions can influence each level differently [2,5]. A central unresolved issue is therefore whether peptides prioritized for high predicted antioxidant potential retain activity across chemical and cellular systems, or whether these readouts capture distinct dimensions of antioxidant function.

Short-peptide antioxidant activity is strongly sequence-dependent, making sequence-aware selection and multilevel evaluation essential for identifying biologically effective candidates. Peptide length, residue composition and position, charge distribution, and local chemical environment influence both redox reactivity and molecular recognition [2,6,7]. Histidine-containing sequences can support proton/electron transfer and metal coordination through the imidazole group, whereas acidic and polar residues can alter electrostatic interactions, hydrogen bonding, solubility, and target recognition [6,7]. These effects depend on residue context rather than composition alone. Peptides with related compositions may therefore respond differently when residue order changes, and the same sequence can rank differently across DPPH, ABTS, and cellular assays [5,6,8]. Such assay-dependent patterns indicate that no single sequence descriptor or chemical endpoint adequately represents overall antioxidant efficacy. It remains important to determine whether high-scoring tripeptides and tetrapeptides display divergent activity profiles and whether such differences can be linked to sequence-specific structural features.

*Apostichopus japonicus* is a protein-rich marine resource with substantial potential for bioactive-peptide discovery, yet its short-peptide sequence space has not been comprehensively explored at the proteome scale. Sea cucumber peptides exhibit antioxidant and other biological activities, and enzymatic hydrolysis remains a major route for releasing bioactive sequences [1,9]. Peptidomic analysis of *A. japonicus* hydrolysates showed that recovered peptide profiles vary markedly with hydrolysis conditions and protease selection [10], while bioinformatics studies have identified numerous potential bioactive precursors in sea cucumber proteins and processing by-products [11]. Antioxidant activity has been reported for an *A. japonicus*-derived peptide in vivo [12], and recent studies have identified active peptides from fermented sea cucumber intestine, *A. japonicus* collagen, and co-fermented *A. japonicus* body-wall proteins [13,14,15]. A sea cucumber-derived tetrapeptide further illustrates the functional potential of very short sequences [16]. However, these workflows largely remain hydrolysate-first, beginning with experimentally generated, fractionated, fermented, or mass-spectrometry-detected peptide pools. A proteome-first strategy can instead interrogate short-peptide sequence space before experimental recovery and thereby broaden candidate discovery. Whether virtual hydrolysis of the *A. japonicus* proteome can systematically uncover antioxidant tripeptides and tetrapeptides for multilevel validation therefore remains unresolved.

Machine-learning prediction and molecular modeling can expand antioxidant-peptide discovery beyond experimentally accessible peptide pools, but computational ranking is not equivalent to biological efficacy. Data-driven approaches, including de novo peptide generation and activity prediction, can prioritize candidates across sequence spaces that are impractical to test exhaustively [17], and virtual screening combined with cellular validation has successfully identified antioxidant peptides from complex food proteins [8]. Molecular docking is frequently used to assess peptide recognition of Kelch-like ECH-associated protein 1 (Keap1), with hydrogen bonding and other non-covalent contacts in the Kelch domain proposed to contribute to peptide-mediated redox-response modulation [5]. Molecular dynamics (MD) simulations add information on receptor stability, conformational sampling, solvent exposure, and persistence of intermolecular contacts over time [18]. Nevertheless, prediction scores, docking scores, receptor-level stability metrics, and cellular protection measure different properties and should not be treated as interchangeable surrogates of antioxidant efficacy. A key question is therefore whether high-ranking peptides exhibit persistent, sequence-dependent Keap1 interactions during MD simulations and whether those computational characteristics correspond to chemical radical-scavenging and cellular-protective outcomes.

The Keap1/Nrf2-associated antioxidant system provides a relevant framework for examining how peptide–protein interactions may relate to cellular redox protection, but pathway-level claims require evidence beyond total protein abundance. Keap1 regulates the stability and availability of nuclear factor erythroid 2-related factor 2 (Nrf2), which controls antioxidant-response genes including heme oxygenase-1 (HO-1), and food-derived peptides have been reported to influence Keap1/Nrf2-related responses in oxidative-stress models [3,4,5,19]. However, changes in total Keap1, Nrf2, or HO-1 do not alone establish canonical pathway activation, which additionally requires evidence such as Nrf2 nuclear translocation or direct disruption of the Keap1–Nrf2 interaction. Against this background, the present study established a proteome-scale workflow integrating virtual trypsin/pepsin hydrolysis, two independent antioxidant-prediction models, consensus ranking, toxicity screening, and sequence-diversity-informed candidate selection to discover and experimentally validate previously uncharacterized antioxidant short peptides from *A. japonicus*. Selected peptides were evaluated using DPPH and ABTS radical-scavenging assays, a D-galactose-induced HepG2 cellular-injury model, molecular docking with the Keap1 Kelch domain, 100 ns MD simulations, and Western blot analysis of Keap1, Nrf2, and HO-1. We hypothesized that *A. japonicus*-derived short peptides could act through both direct chemical antioxidant effects and cellular antioxidant responses, and specifically tested whether predicted activity, radical-scavenging performance, peptide–Keap1 interaction characteristics, and cellular cytoprotection converged on the same candidates or instead represented related but non-equivalent layers of antioxidant performance.

## 2. Materials and Methods

### 2.1. Sea Cucumber Proteome Retrieval and In Silico Enzymatic Hydrolysis

The reference proteome of the sea cucumber *Apostichopus japonicus* (UniProt Proteome ID: UP000230750; NCBI Taxonomy ID: 307972) was retrieved from UniProt in FASTA format on 21 June 2026 [20]. The dataset contained 30,032 protein entries associated with genome assembly GCA_002754855.1. In silico enzymatic hydrolysis was performed using a custom Python 3.9 script with Biopython SeqIO for sequential FASTA parsing.

Trypsin cleavage was simulated at the C-terminal side of Arg (R) and Lys (K), except when Pro (P) occupied the P1′ position. Pepsin cleavage was simulated on both sides of Phe (F), Tyr (Y), Trp (W), and Leu (L), with cleavage suppressed when Pro was adjacent to the corresponding cleavage site, following the cleavage pattern adopted from BIOPEP-UWM [21]. Complete cleavage was assumed and missed cleavages were not considered. Only tripeptides and tetrapeptides composed exclusively of the 20 standard proteinogenic amino acids were retained. Duplicate peptide sequences were removed globally, and the resulting unique peptides were exported as a CSV file for subsequent activity prediction.

### 2.2. Antioxidant Peptide Prediction, Ensemble Ranking, and Toxicity Screening

Two publicly available antioxidant peptide prediction models, Pred5AOP [22] and Multi-AOP [23], were deployed locally using the original source code, preprocessing procedures, model architectures, and pre-trained parameters without retraining or modification. Pred5AOP used RDKit-derived Morgan fingerprints (radius 2; 2048 bits) generated from peptide molecular representations, whereas Multi-AOP used its original multi-view sequence- and structure-based feature framework. Model inference was performed using PyTorch (version 2.3), and peptides that could not be processed by either original implementation were excluded from the corresponding prediction results.

For peptides successfully processed by both models, a consensus ensemble score was calculated as the arithmetic mean of the two model outputs and used as a relative ranking index:

Sensemble = (SPred5AOP + SMulti-AOP)/2

The ranked candidates were further evaluated using the standalone implementation of ToxinPred3.0 [24], employing Model 2 with the default classification cutoff of 0.38. Peptides classified as “Toxin” were excluded, whereas “Non-Toxin” peptides were retained for subsequent experimental evaluation. Prediction outputs and data integration were handled using custom Python scripts and Pandas.

### 2.3. Peptide Synthesis and Radical-Scavenging Assays

Selected candidate antioxidant peptides were custom synthesized by Sangon Biotech (Shanghai) Co., Ltd. (Shanghai, China). Peptide purity was assessed by analytical HPLC, and molecular identity was verified by electrospray ionization mass spectrometry (ESI-MS). The manufacturer-reported HPLC purities of DHE, HDHE, DDHN, EHED, and QHDE were 95.976%, 99.189%, 97.308%, 95.829%, and 97.834%, respectively. The corresponding HPLC chromatograms and mass spectra are provided in the Supplementary Materials. Peptide concentrations were calculated from the dry-product mass and theoretical molecular weight and were corrected according to the manufacturer-reported HPLC purity. Peptides were dissolved in deionized water before use. DPPH and ABTS radical-scavenging activities were measured using 96-well plate assays adapted from Zhang et al. [22], with glutathione (GSH; Solarbio, Beijing, China) at 2 mM as the positive control. The corresponding GSH positive-control radical-scavenging data are shown in Figure S1A.

For the DPPH assay, peptide solutions were prepared at twice the target final concentrations. Peptide solution (100 μL) was mixed with an equal volume of 0.1 mM DPPH in ethanol, giving final peptide concentrations of 0.125, 0.25, 0.5, 1.0, and 2.0 mM. The reaction mixtures remained visually homogeneous and clear, with no visible precipitation, phase separation, or turbidity during the 30 min incubation at 25 °C in the dark. Absorbance was then measured at 517 nm. Deionized water was used instead of peptide solution in the control group, and an additional blank was prepared by replacing the DPPH solution with ethanol. DPPH radical-scavenging activity was calculated as [(Ac − As)/Ac] × 100, where As and Ac represent the absorbances of the sample and control groups, respectively.

For preparation of ABTS•+, 15 mg of ABTS was dissolved in 4 mL of deionized water (approximately 6.83 mM), and 10 mg of K_2_S_2_O_8_ was dissolved in 35 mL of deionized water (approximately 1.06 mM). Equal volumes (0.2 mL each) of the two solutions were mixed and incubated for 16 h at room temperature in the dark. The resulting solution was diluted 40-fold with 1× PBS prepared from commercial 10× PBS buffer (Sangon Biotech, Shanghai, China; Order No. E607081). The 1× PBS contained 137 mM NaCl, 2.7 mM KCl, 10 mM Na_2_HPO_4_·12H_2_O, and 1.8 mM KH_2_PO_4_ (pH 7.4 ± 0.1) and contained no CaCl_2_ or MgCl_2_. The absorbance of a freshly prepared 40-fold diluted ABTS•+ working solution was 0.434 ± 0.010 at 405 nm before sample addition. Peptide solutions were prepared at four times the target final concentrations. Peptide solution (50 μL) was mixed with 150 μL of ABTS•+ working solution, giving final peptide concentrations of 0.125, 0.25, 0.5, 1.0, and 2.0 mM, and the mixtures were incubated for 10 min at 25 °C in the dark. Absorbance was measured at 405 nm to monitor the short-wavelength visible absorption of ABTS•+, which exhibits a strong absorption band near 415 nm; 405 nm has also been used for microplate-based ABTS antioxidant assays [25,26]. To evaluate potential peptide-specific absorbance at this wavelength, an additional control experiment was performed during revision using the same final peptide concentrations as in the ABTS assay but without ABTS•+. A water/PBS vehicle blank was measured in parallel. The vehicle blank showed an absorbance of 0.079 ± 0.012 at 405 nm, and the peptide-only absorbance values largely overlapped the vehicle background and showed no systematic concentration-dependent increase, indicating only a limited contribution of peptide-specific absorbance under the assay conditions. ABTS radical-scavenging activity was calculated as [(Ac − As)/Ac] × 100, where As and Ac represent the absorbances of the sample and control groups, respectively.

### 2.4. HepG2 Cell Culture and Cytotoxicity Assay

HepG2 cells were purchased from Procell Life Science & Technology Co., Ltd. (Wuhan, China) and cultured in Dulbecco’s modified Eagle’s medium (DMEM; Sangon Biotech (Shanghai) Co., Ltd., Shanghai, China) supplemented with 10% fetal bovine serum and 1% penicillin–streptomycin at 37 °C in a humidified atmosphere containing 5% CO2. Cells in the logarithmic growth phase were used for experiments, following Zhang et al. [3] with modifications.

For cytotoxicity assessment, cells were seeded in 96-well plates at 5 × 10^3^ cells/well for 24 h and then treated with peptides at 0.125, 0.25, 0.5, 1, or 2 mM for an additional 24 h. Cell viability was evaluated using CCK-8 reagent (Sangon Biotech (Shanghai) Co., Ltd., Shanghai, China), added at 10% (*v*/*v*) of the culture-medium volume. After incubation at 37 °C for 2 h, absorbance was measured at 450 nm.

### 2.5. D-Galactose-Induced Cellular Injury and Cytoprotective Assay

To establish the cell-injury model, HepG2 cells were exposed to D-galactose (Solarbio, Beijing, China) at 100, 200, 300, 400, or 500 mM for 24 h. Cell viability was determined by CCK-8, and 200 mM D-galactose for 24 h was selected for the subsequent protection assay. For cytoprotection experiments, cells were pretreated with peptides (0.125, 0.25, 0.5, 1.0, or 2.0 mM) for 24 h, followed by replacement with fresh medium containing 200 mM D-galactose for another 24 h. The normal control, D-galactose model, peptide-treated, and 2 mM GSH-treated positive-control groups were included. The corresponding GSH positive-control cytoprotective data are shown in Figure S1B.

For all CCK-8 measurements, reagent was added at 10% (*v*/*v*) of the culture-medium volume and incubated for 2 h at 37 °C before absorbance measurement at 450 nm. Cell viability was calculated as [(As − Ab)/(Ac − Ab)] × 100, where As is the absorbance of the treatment group, Ac is the untreated cell control, and Ab is the blank well without cells.

### 2.6. Western Blot Analysis

After the indicated treatments, HepG2 cells were harvested and lysed to obtain total cellular protein. For Western blot analysis, each of the five peptides was used at 2 mM, with the remaining treatment conditions following the cytoprotection protocol described above. Protein concentrations were determined using a bicinchoninic acid (BCA) assay. Equal amounts of protein (40 µg per lane) were separated on 10% SDS-PAGE gels prepared using a one-step rapid gel preparation kit (Epizyme Biotech, Shanghai, China) at a constant voltage of 150 V. Proteins were transferred to membranes at a constant current of 200 mA for 90 min. Membranes were blocked with rapid blocking buffer (Epizyme Biotech, Shanghai, China). Primary antibodies against KEAP1 (Cat. No. 10503-2-AP), HO-1 (Cat. No. 10701-1-AP), and β-actin (Cat. No. 66009-1-Ig) were purchased from Proteintech Group, whereas the NRF2 primary antibody (Cat. No. 12721T) was purchased from Cell Signaling Technology, Danvers, MA, USA. Membranes were incubated with the primary antibodies overnight at 4 °C, followed by incubation with the corresponding secondary antibodies at 37 °C for 1 h. Immunoreactive bands were visualized by chemiluminescence using a ChemiDoc Imaging System (Bio-Rad Laboratories, Inc., Hercules, CA, USA). Band densitometry was performed using the Gel Analyzer function in ImageJ(version 1.54i). For each target-protein region, a rectangular lane-selection window was selected to be as narrow as practicable while encompassing the complete immunoreactive band together with only a small amount of adjacent background, and the same window dimensions were applied to the corresponding lanes. Lane intensity profiles were generated using Analyze → Gels → Plot Lanes. A consistent baseline was defined for the corresponding protein peak, and the integrated peak area was quantified using the Wand Tool. Relative protein expression was determined by normalization of the target-protein peak area to the corresponding β-actin signal. Three independent biological replicates were analyzed (*n* = 3).

### 2.7. Molecular Docking of Peptides with KEAP1

The crystal structure of KEAP1 was obtained from the Protein Data Bank (PDB ID: 2FLU) [27]. The original Nrf2 peptide and crystallographic water molecules were removed, hydrogen atoms were added, and the receptor was converted to PDBQT format before docking. Three-dimensional structures of the peptides were constructed using Avogadro. KEAP1 was used as the receptor and each peptide as a ligand for molecular docking using AutoDock Vina version 1.2.0 [28]. The docking search space was centered at x = 17.739, y = 18.750, and z = 9.974 Å, with dimensions of 61.878 × 64.799 × 62.406 Å along the x, y, and z axes, respectively. A grid spacing of 0.375 Å was used, and the exhaustiveness parameter was set to 32. Docking calculations were performed using eight CPU threads. For each peptide, the lowest-energy docking pose was selected as the starting structure for subsequent molecular dynamics simulations.

### 2.8. Molecular Dynamics System Preparation and Equilibration

All molecular dynamics (MD) systems were prepared using AmberTools 22. The apo KEAP1 structure and peptide–KEAP1 complexes were processed with pdb4amber and Reduce. The protein was described using the ff19SB force field [29]. For ligand components requiring nonstandard parameters, AM1-BCC partial charges and GAFF2 parameters were generated using the antechamber module in AmberTools [30,31]. Each system was solvated in an explicit OPC water box [32] with a 15.0 Å buffer and neutralized with counterions using OPC-compatible Li/Merz 12-6 ion parameters (frcmod.ion- slm_126_opc, accessed on 15 June 2026) [33].

Each system underwent two 5000-step minimization stages, first with a 10.0 kcal mol^−1^ Å^−2^ solute restraint and then without restraints. Systems were heated from 0 to 310.15 K over 200 ps under NVT conditions using a Langevin thermostat (collision frequency 2.0 ps^−1^), followed by 200 ps of restrained NPT density equilibration at 310.15 K and 1.0 atm and a further 500 ps of unrestrained NPT equilibration.

### 2.9. Production Molecular Dynamics Simulations

Production MD simulations were performed for 100 ns under the NPT ensemble at 310.15 K and 1.0 atm using the GPU-accelerated pmemd.cuda engine. Long-range electrostatics were treated using particle mesh Ewald, with a 10.0 Å cutoff for short-range nonbonded interactions. Bonds involving hydrogen atoms were constrained using SHAKE, permitting a 2.0 fs time step. Coordinates were saved every 100 ps, yielding 1000 frames per trajectory.

### 2.10. Trajectory Processing and Structural Analysis

Trajectory processing and structural analyses were conducted using CPPTRAJ in AmberTools 22 [34]. Water molecules and counterions were removed before analysis. Global structural stability and flexibility of Keap1 were evaluated from protein Cα atoms using root-mean-square deviation (RMSD), root-mean-square fluctuation (RMSF), and radius of gyration (Rg). Solvent-accessible surface area (SASA) was calculated using the surf command, and protein secondary structure was characterized using the CPPTRAJ secstruct module based on DSSP assignments.

### 2.11. Principal Component Analysis and Free-Energy Landscape

Principal component analysis (PCA) was performed on the 100 ns trajectories of apo KEAP1 and the five peptide–KEAP1 complexes using CPPTRAJ [34]. The covariance matrix of atomic positional fluctuations was diagonalized, and PC1 and PC2 were used to describe the dominant conformational motions. The PC1–PC2 space was divided into a 50 × 50 grid and smoothed using Gaussian broadening (0.5). The relative free-energy landscape (FEL) at 310.15 K was calculated as:

ΔG = −RT ln(P/Pmax)

where P is the probability of a conformational state and Pmax is the maximum probability. The global minimum was normalized to 0 kcal/mol. The connected region within ΔG ≤ 0.5 kcal/mol was defined as the principal low-energy basin, and the real trajectory snapshot closest to its probability-weighted center was selected as the representative conformation.

### 2.12. Hydrogen-Bond Occupancy Analysis

Intermolecular hydrogen bonds between KEAP1 and each peptide were analyzed from solvent-stripped trajectories using MDAnalysis [35]. Hydrogen bonds in both donor directions were identified using a donor–acceptor distance of ≤3.0 Å and a D–H···A angle of ≥150°. Occupancy was calculated as the percentage of the 1000 trajectory frames in which each donor–acceptor residue pair was present after removal of duplicate atom-level records within individual frames. For visualization, the 10 hydrogen-bond pairs with the highest occupancies were displayed, while the complete interaction dataset was retained for supplementary reporting.

### 2.13. Statistical Analysis

DPPH and ABTS radical-scavenging assays and CCK-8-based cell experiments were performed with *n* = 4, whereas Western blot analyses were performed using three independent biological replicates (*n* = 3). Data are presented as mean ± standard deviation (SD) unless otherwise indicated. Statistical analyses and graphical presentation were performed using GraphPad Prism 10.0. Normality and homogeneity of variance were assessed using the Shapiro–Wilk and Levene’s tests, respectively. DPPH and ABTS radical-scavenging activities were analyzed separately using ordinary two-way ANOVA with peptide identity and concentration as categorical factors, including their interaction. Tukey’s multiple-comparisons test was subsequently used to compare all five concentrations within each peptide. Other group comparisons were analyzed by one-way ANOVA followed by Dunnett’s multiple-comparisons test as appropriate. *p* < 0.05 was considered statistically significant; significance was denoted as ns (*p* > 0.05), * (*p* ≤ 0.05), ** (*p* ≤ 0.01), *** (*p* ≤ 0.001), and **** (*p* ≤ 0.0001).

## 3. Results and Discussion

### 3.1. In Silico Screening and Prioritization of Potential Antioxidant Peptides

Starting from the 30,032-protein *Apostichopus japonicus* reference proteome, virtual trypsin/pepsin hydrolysis followed by length- and composition-based filtering and global deduplication yielded 37,479 unique eligible tripeptide and tetrapeptide sequences, including 3035 tripeptides and 34,444 tetrapeptides. All 37,479 sequences were successfully scored by both Multi-AOP and Pred5AOP, and the arithmetic mean of the two model outputs was used as a consensus score for relative candidate ranking. Subsequent ToxinPred3.0 screening retained 27,396 peptides predicted to be non-toxic, corresponding to 73.1% of the dual-model-scored sequence set. The ten highest-ranked non-toxic candidates are summarized in Table 1, with consensus scores ranging from 0.945 to 0.965. DHE ranked first with a consensus score of 0.965, followed by DDHN and HDHE at 0.961. This multistage funnel reduced the proteome-derived short-peptide sequence space to a focused pool of high-ranking candidates for sequence-diversity-informed selection and experimental validation.

Both prediction models assigned high antioxidant scores to the leading candidates, although sequence-specific differences in score magnitude were evident. EHED received a higher Pred5AOP than Multi-AOP score, whereas QHDE showed the opposite pattern, consistent with the distinct molecular representations and model architectures used by the predictors. The consensus score was therefore used as a relative prioritization index rather than an absolute probability, reducing dependence on any single model.

Final candidate selection was not based solely on numerical rank. To avoid advancing a set of highly similar sequences, high-scoring candidates were selected to maximize sequence and residue-pattern diversity for experimental comparison. DHE represented the highest-ranked compact tripeptide; HDHE a histidine-rich sequence; DDHN a peptide with consecutive acidic residues; EHED a distinct acidic/histidine arrangement; and QHDE a glutamine-containing sequence with additional hydrogen-bonding potential.

Accordingly, DHE, HDHE, DDHN, EHED, and QHDE were synthesized for validation. To the best of our knowledge, these sequences have not previously been reported as standalone antioxidant peptides derived from *A. japonicus*, supporting their further evaluation as newly identified antioxidant candidates. Prediction was used here as a prioritization layer rather than a surrogate for experimental performance, enabling direct comparison of whether similarly high-scoring but structurally distinct peptides retained their rank across chemical, cellular, and molecular-interaction readouts.

Notably, although none of the five experimentally selected peptides contained cysteine, Cys-containing sequences were not excluded from the screening workflow, as all 20 standard proteinogenic amino acids were permitted during virtual hydrolysis and sequence filtering. Among the 37,479 dual-model-scored candidates prior to toxicity filtering, 7651 (20.41%) contained at least one Cys residue. Within this full pre-toxicity-screening set, no Cys-containing peptide occurred among the top 50 consensus-ranked candidates, only one occurred among the top 100, and three were present among the top 1% (375 sequences); the highest-ranked Cys-containing peptide, DHDC, ranked 70th with a consensus score of 0.9158. Thus, the absence of Cys among the experimentally selected peptides reflected the outcome of the present prediction-based prioritization rather than an a priori sequence restriction. Importantly, antioxidant activity of short peptides is not exclusively dependent on Cys; histidine as well as acidic and polar residues can also contribute to redox activity through sequence-dependent proton/electron-transfer and chemical interactions [6,7], consistent with the measurable DPPH and ABTS radical-scavenging activities observed for all five Cys-free peptides in this study. Given the well-established redox reactivity of the cysteine thiol group, Cys-containing short peptides remain an important complementary sequence class for future targeted investigation.

### 3.2. In Vitro Antioxidant Activities of Selected Sea Cucumber-Derived Peptides

**Figure 1:**
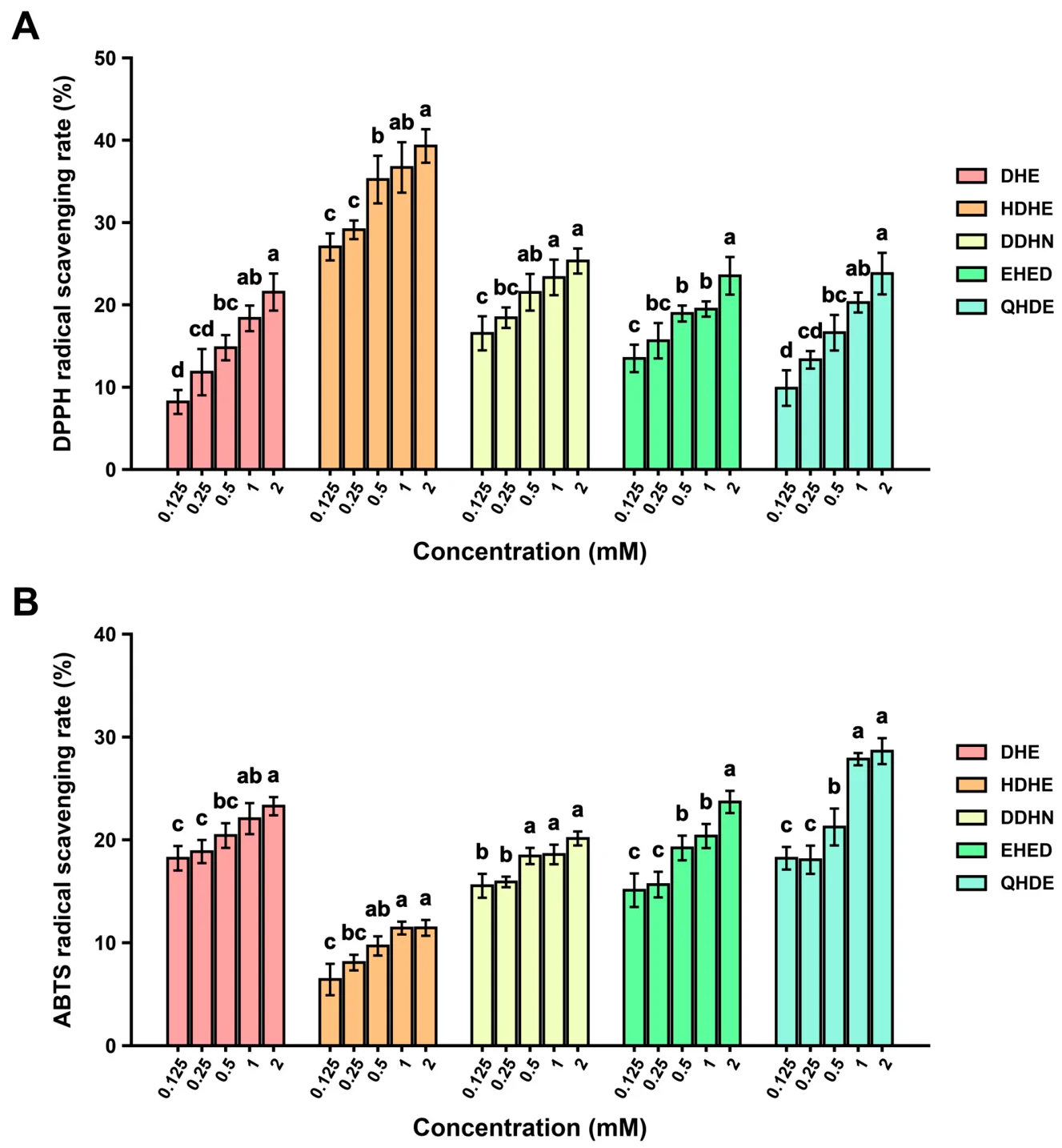
DPPH and ABTS radical-scavenging activities of selected sea cucumber-derived peptides at different concentrations. (**A**) DPPH and (**B**) ABTS radical-scavenging activities of DHE, HDHE, DDHN, EHED, and QHDE at final concentrations of 0.125, 0.25, 0.5, 1.0, and 2.0 mM. Bars are grouped by peptide, with the five concentrations shown from left to right within each group. Data are presented as mean ± SD (*n* = 4 per condition). Each assay was analyzed separately using ordinary two-way ANOVA with peptide identity and concentration as factors, including their interaction, followed by Tukey’s multiple-comparisons test among concentrations within each peptide. Within the same peptide and panel, bars sharing no lowercase letter differ significantly at Tukey-adjusted *p* ≤ 0.05, whereas bars sharing at least one letter do not differ significantly. Letter assignments are independent across peptides and panels.

DPPH radical-scavenging activity was significantly affected by peptide identity and concentration (both *p* < 0.0001), whereas the peptide × concentration interaction was not significant (*p* = 0.3014). ABTS radical-scavenging activity showed significant effects of peptide identity, concentration, and their interaction (all *p* < 0.0001). Within-peptide Tukey comparisons showed significantly greater activity at 2.0 mM than at 0.125 mM for all five peptides in both assays, although not all successive concentration increments resulted in significant increases, as indicated by the overlapping lowercase-letter groupings in Figure 1. HDHE displayed the highest DPPH activity, increasing from approximately 27% at 0.125 mM to nearly 40% at 2 mM, whereas DDHN showed intermediate activity and DHE, EHED, and QHDE were lower. Thus, computational prioritization successfully enriched for peptides with measurable direct radical-scavenging activity, although the experimental rank order differed from the consensus prediction. Similar sequence-dependent DPPH and ABTS activities have been reported for other short food-derived peptides [6,36].

The ABTS assay produced a different activity hierarchy (Figure 1B). QHDE showed a pronounced concentration-dependent increase and the highest ABTS activity at 1 and 2 mM, whereas HDHE, despite leading the DPPH assay, remained comparatively weak against ABTS. These divergent rankings show that radical-scavenging performance is reaction-system dependent, consistent with differences in radical accessibility and electron- or hydrogen-transfer behavior reported for antioxidant peptides [6,7]. At 2 mM, the GSH positive control showed approximately 63.2% DPPH and 81.2% ABTS radical-scavenging activity (Figure S1A), providing a reference antioxidant response for both assay systems.

The high DPPH activity of HDHE may be partly related to its two histidine residues, whose imidazole groups can participate in proton/electron transfer and radical stabilization. Its weaker ABTS response, however, shows that histidine abundance alone does not determine antioxidant performance. Conversely, QHDE suggests that the arrangement of polar and acidic residues also contributes to radical-specific reactivity. Antioxidant activity therefore reflects residue composition, position, local environment, and peptide conformation rather than a single sequence feature [6,7]. Together, the DPPH and ABTS data establish chemical antioxidant activity while also illustrating the paper’s central distinction: predicted potential and assay-specific radical scavenging are related, but they are not equivalent measures of peptide performance.

### 3.3. Cytocompatibility and Cytoprotective Effects of Selected Peptides Under D-Galactose-Induced Cellular Injury

**Figure 2:**
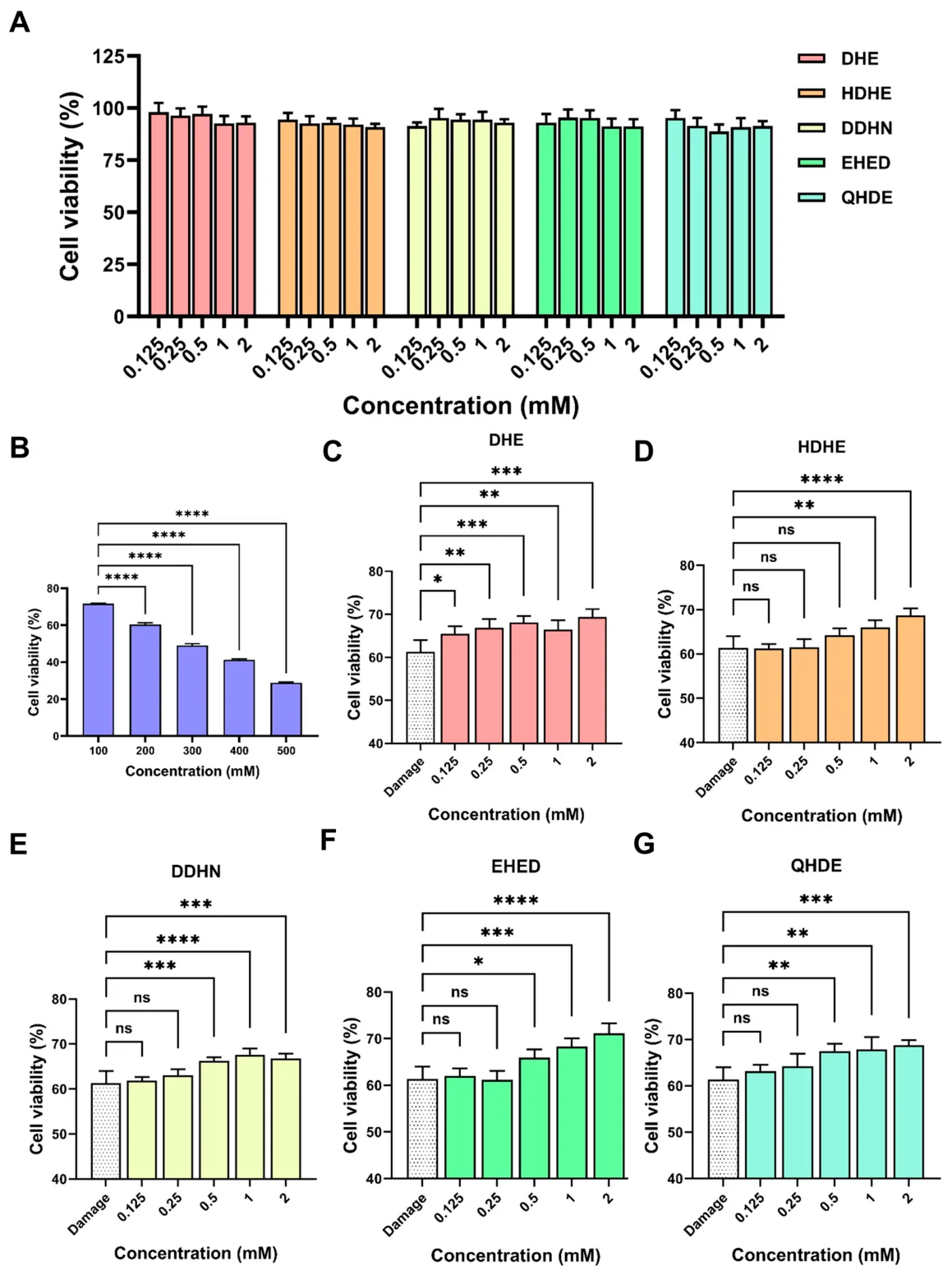
Cytocompatibility and cytoprotective effects of selected sea cucumber-derived peptides under D-galactose-induced cellular injury. (**A**) Effects of DHE, HDHE, DDHN, EHED, and QHDE (0.125–2 mM) on HepG2 cell viability after 24 h of treatment. (**B**) Effects of D-galactose (100–500 mM) exposure for 24 h on HepG2 cell viability. Cell viability was normalized to the untreated control group, which was defined as 100%. (**C**–**G**) Protective effects of DHE (**C**), HDHE (**D**), DDHN (**E**), EHED (**F**), and QHDE (**G**) against cellular injury induced by 200 mM D-galactose. Cells were pretreated with the indicated peptide concentrations for 24 h and subsequently exposed to 200 mM D-galactose for an additional 24 h. Data are presented as mean ± SD from three independent experiments (*n* = 4). In (**B**), statistical significance was determined relative to the untreated control group; in (**C**–**G**), statistical significance was determined relative to the D-galactose damage group. ns, *p* > 0.05; * *p* ≤ 0.05; ** *p* ≤ 0.01; *** *p* ≤ 0.001; and **** *p* ≤ 0.0001. GSH (2 mM) was included as a positive control, and the corresponding cytoprotective data are shown in Figure S1B.

The five peptides showed good cytocompatibility in HepG2 cells, with viability generally remaining above 90% across the tested concentration range (Figure 2A). D-galactose exposure caused a concentration-dependent loss of viability, from approximately 71% at 100 mM to below 30% at 500 mM (Figure 2B). Because 200 mM D-galactose reduced viability to approximately 60%, this condition was selected as an intermediate cellular-injury model. At excessive concentrations, D-galactose can increase ROS generation and thereby promote oxidative stress, leading to cellular damage and impaired cell viability [37]. Accordingly, the present model is interpreted primarily as D-galactose-induced cellular injury, with its oxidative-stress context supported by previous studies rather than by direct intracellular ROS measurement in this experiment.

Pretreatment with the selected peptides alleviated D-galactose-induced loss of cell viability to different extents (Figure 2C–G). DHE significantly improved viability across the tested concentrations, HDHE was protective mainly at higher concentrations, and DDHN, EHED, and QHDE showed clearer concentration-dependent effects from intermediate or high concentrations. EHED at 2 mM restored viability to approximately 71%, the greatest recovery among the tested peptide treatments. Comparable cytoprotective effects have been reported for food-derived antioxidant peptides in D-galactose- and other oxidative stress-associated HepG2 models [3,4,38,39]. Consistently, 2 mM GSH significantly increased the viability of D-galactose-treated HepG2 cells from approximately 60% to 78%, while GSH treatment alone maintained viability near 100% (Figure S1B), supporting the responsiveness of the cellular protection model to a reference antioxidant.

Cellular protection did not reproduce the chemical radical-scavenging hierarchy. HDHE led the DPPH assay but produced only moderate cytoprotection, whereas EHED showed modest direct radical scavenging yet the greatest recovery of D-galactose-treated cells. This divergence indicates that cellular efficacy cannot be inferred from a cell-free radical assay alone and may additionally depend on peptide stability, cellular interactions, and modulation of endogenous antioxidant responses. Antioxidant peptides have been reported to protect HepG2 cells through Keap1/Nrf2-associated mechanisms [3,4,38], motivating the subsequent molecular and protein-expression analyses.

### 3.4. Molecular Docking Analysis of Peptide–Keap1 Interactions

**Figure 3:**
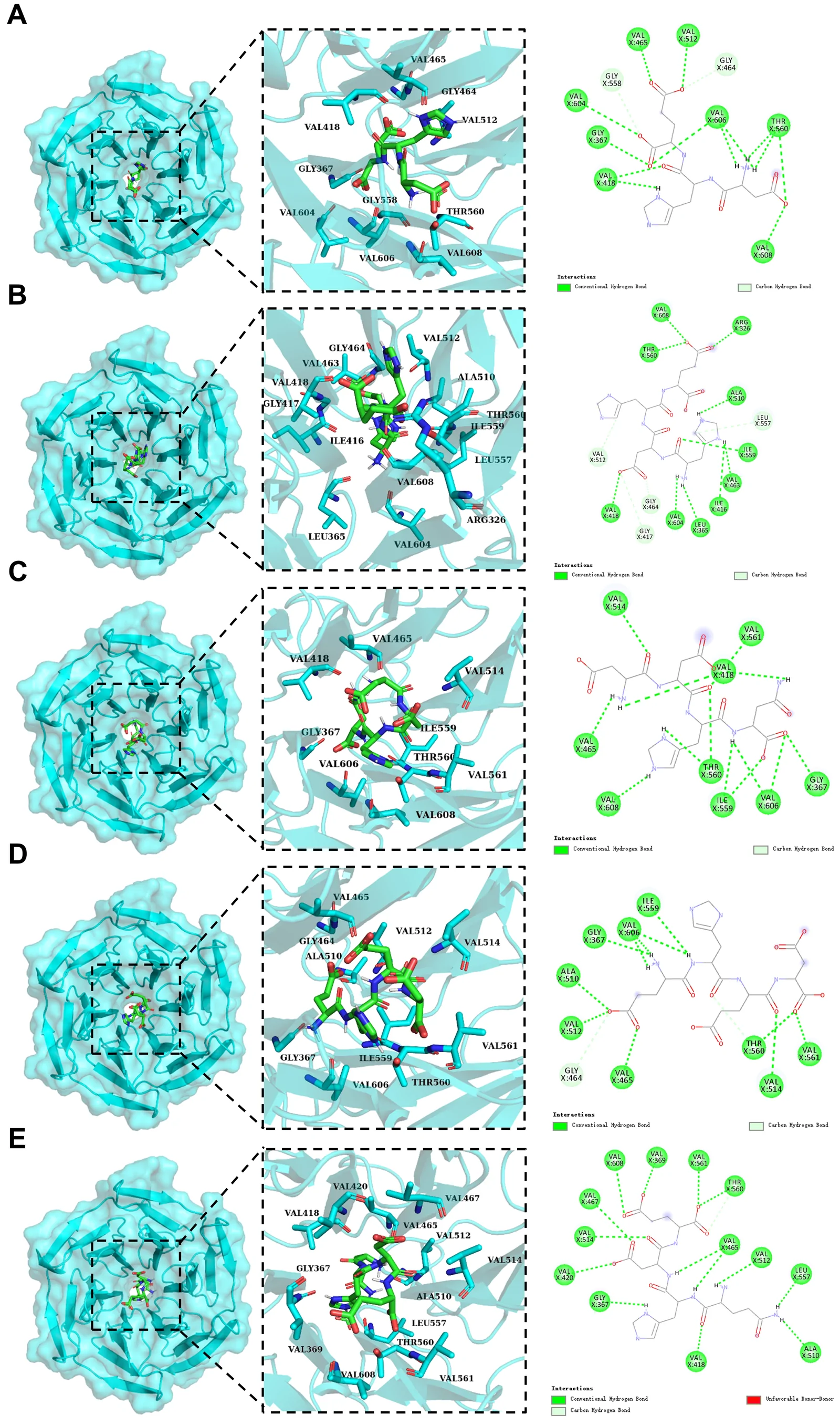
Molecular docking modes and intermolecular interactions of selected peptides with Keap1. (**A**–**E**) Representative docking poses of DHE, HDHE, DDHN, EHED, and QHDE within the Keap1 Kelch domain, respectively. Specifically, (**A**) DHE–Keap1, (**B**) HDHE–Keap1, (**C**) DDHN–Keap1, (**D**) EHED–Keap1, and (**E**) QHDE–Keap1. Each panel includes an enlarged binding-pocket view and a two-dimensional interaction diagram showing key peptide–Keap1 contacts.

Molecular docking was performed using AutoDock Vina v1.2.0 to investigate potential interactions between the selected peptides and the Keap1 Kelch domain. The five peptides exhibited Vina docking scores ranging from −7.861 to −9.031 kcal/mol. HDHE showed the most favorable docking score (−9.031 kcal/mol), followed by QHDE (−8.896 kcal/mol), DDHN (−8.158 kcal/mol), DHE (−8.024 kcal/mol), and EHED (−7.861 kcal/mol). As shown in Figure 3, all peptides were accommodated within the selected Keap1 binding cavity and formed multiple predicted non-covalent contacts with surrounding residues. Hydrogen bonding was a major predicted interaction type, with residues including Gly367, Val418, Val465, Thr560, and Val608 repeatedly participating in peptide contacts. Similar interactions between antioxidant peptides and the Keap1 Kelch domain have been reported in recent studies [18,40,41,42].

Although the five peptides occupied a broadly similar docking region, their predicted interaction patterns differed according to peptide sequence. HDHE formed an extensive predicted contact network and exhibited the most favorable docking score among the five peptides. QHDE also established a broad predicted contact network involving residues such as Gly367, Val418, Val465, Val512, Thr560, and Val608. DDHN, DHE, and EHED displayed distinct hydrogen-bonding patterns involving acidic and polar residues, suggesting that amino acid composition and residue arrangement influenced the predicted modes of peptide–Keap1 recognition [18,40,42].

The docking-score hierarchy did not match the cellular response. EHED had the least favorable Vina score yet produced the greatest recovery of D-galactose-treated HepG2 cells. This mismatch is informative rather than contradictory: docking scores estimate the favorability of static poses, whereas cellular efficacy also depends on dynamic contacts, peptide behavior in the cellular environment, and downstream responses. Molecular dynamics simulations were therefore used to examine receptor structural behavior and time-dependent peptide–Keap1 interactions, providing a distinct layer of evidence rather than a direct proxy for cytoprotection.

### 3.5. Molecular Dynamics Characterization of Peptide–Keap1 Complexes

**Figure 4:**
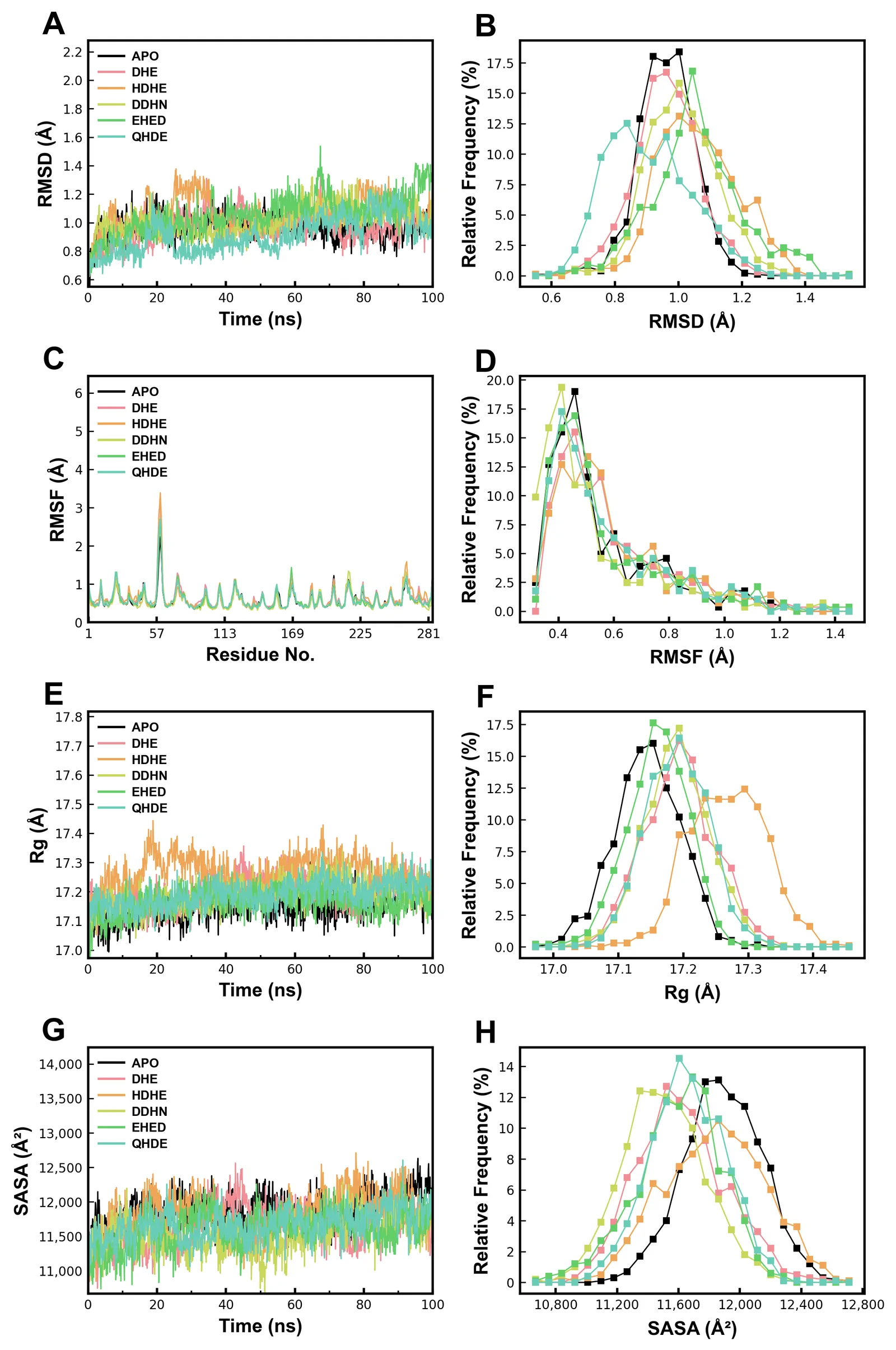
Molecular dynamics analysis of the structural stability and flexibility of peptide–Keap1 complexes. (**A**) Root-mean-square deviation (RMSD) profiles and (**B**) corresponding RMSD distributions of apo Keap1 and the DHE–, HDHE–, DDHN–, EHED–, and QHDE–Keap1 complexes during 100 ns molecular dynamics simulations. (**C**) Root-mean-square fluctuation (RMSF) profiles of Keap1 residues and (**D**) corresponding RMSF distributions. (**E**) Radius of gyration (Rg) profiles and (**F**) corresponding Rg distributions reflecting the overall compactness of the protein. (**G**) Solvent-accessible surface area (SASA) profiles and (**H**) corresponding SASA distributions of the simulated systems. RMSD, root-mean-square deviation; RMSF, root-mean-square fluctuation; Rg, radius of gyration; SASA, solvent-accessible surface area.

To characterize receptor-level structural behavior after peptide introduction, 100 ns MD simulations were performed for apo Keap1 and all five peptide-containing systems. Protein Cα RMSD values remained within a relatively narrow range throughout the trajectories (Figure 4A,B), indicating that none of the systems underwent major global structural disruption. QHDE–Keap1 showed relatively low and concentrated RMSD values, whereas HDHE displayed a transient increase during the early-to-middle stage and EHED somewhat larger fluctuations later in the simulation. These differences indicate system-specific conformational responses without evidence of global receptor destabilization. Because the RMSD calculation was based on protein Cα atoms, it supports Keap1 structural stability rather than continuous peptide occupancy of the binding site; comparable receptor-level stability patterns have been reported for antioxidant peptide–Keap1 systems [18,43,44,45].

The RMSF profiles were also broadly similar among the six systems (Figure 4C,D), showing that peptide presence did not substantially reorganize the overall residue-flexibility pattern of Keap1. The largest local fluctuation occurred around topology residue 57, corresponding to Keap1 residue 381, with the HDHE-containing system showing the greatest variation in this region. Most other residues remained below approximately 1.5 Å. Thus, the dominant differences among systems were localized changes in flexibility rather than widespread alteration of the Keap1 fold.

The radius of gyration remained stable throughout the 100 ns simulations (Figure 4E,F), further supporting preservation of receptor compactness. HDHE–Keap1 showed a modest shift toward higher Rg values than apo Keap1 and the other peptide-containing systems, consistent with a slightly more expanded receptor conformation but not global destabilization. Protein SASA values likewise fluctuated around stable levels without sustained drift (Figure 4G,H). The lower dominant SASA values observed for DDHN–Keap1 relative to apo Keap1 suggest that peptide-dependent conformational sampling altered receptor surface exposure to different extents.

Taken together, RMSD, RMSF, Rg, and SASA describe a globally stable Keap1 receptor with sequence-dependent differences in local flexibility, compactness, and solvent exposure. These receptor-level metrics provide structural context for the peptide-bound systems but do not directly quantify peptide–Keap1 contact persistence. The trajectories were therefore examined further by PCA-based free-energy landscapes and explicit intermolecular hydrogen-bond analysis.

**Figure 5:**
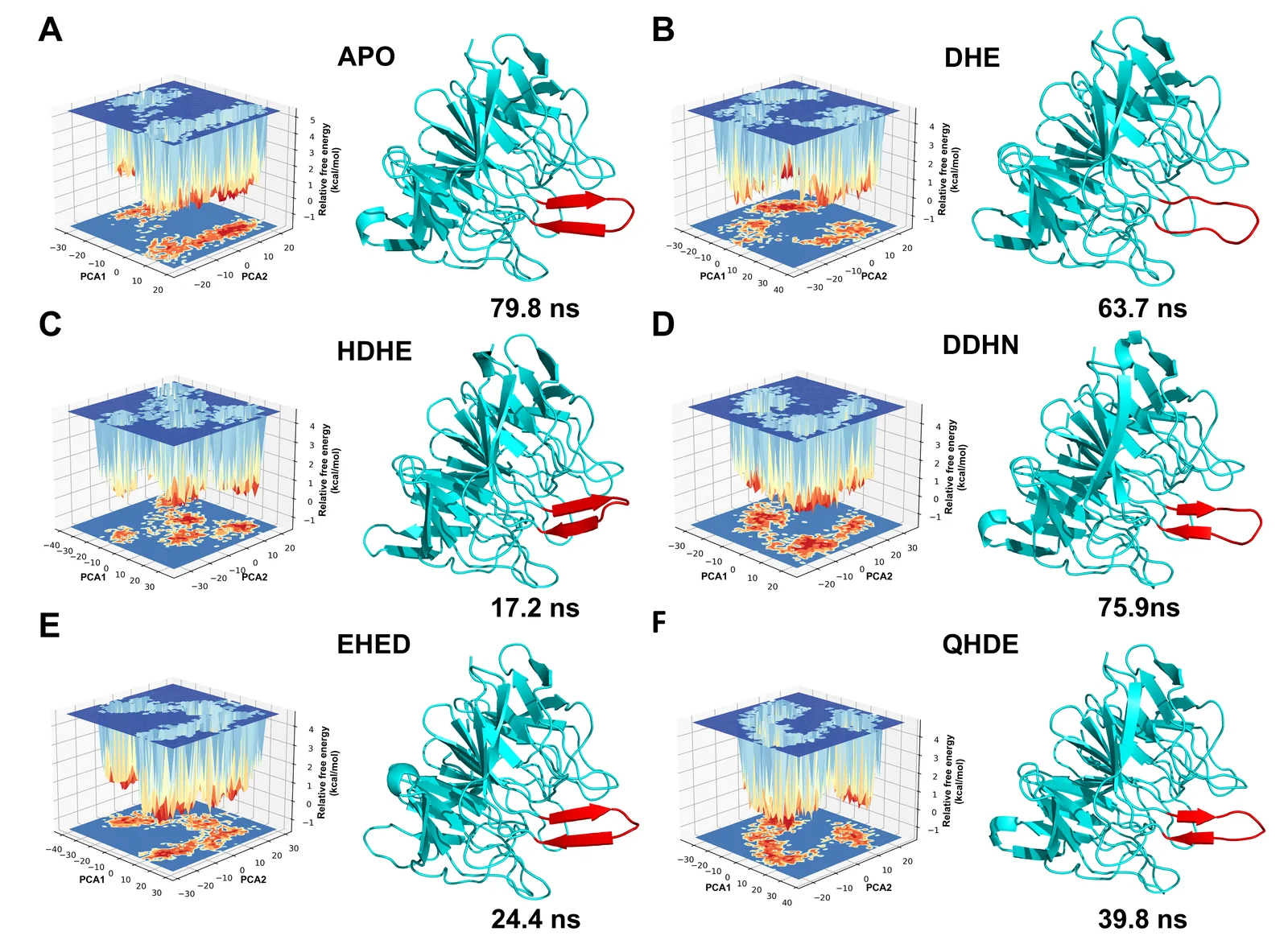
Principal component-based free-energy landscapes and representative low-energy conformations of apo Keap1 and peptide–Keap1 complexes. (**A**) Free-energy landscape of apo Keap1 projected onto PC1 and PC2, with a representative conformation corresponding to the major low-energy state; (**B**) free-energy landscape of the DHE–Keap1 complex and its representative low-energy conformation; (**C**) free-energy landscape of the HDHE–Keap1 complex and its representative low-energy conformation; (**D**) free-energy landscape of the DDHN–Keap1 complex and its representative low-energy conformation; (**E**) free-energy landscape of the EHED–Keap1 complex and its representative low-energy conformation; (**F**) free-energy landscape of the QHDE–Keap1 complex and its representative low-energy conformation. In the free-energy maps, warm colors (red/orange) denote lower relative free-energy regions, whereas blue denotes higher relative free-energy regions.

PCA-based free-energy landscape analysis was used to characterize the dominant conformational states sampled during the MD trajectories (Figure 5). Apo Keap1 and all five peptide-containing systems populated one or more low-energy basins within their PC1–PC2 projections, indicating preferential sampling of particular conformational states within the dominant motions captured by each analysis. The number, breadth, and distribution of these basins differed among systems, demonstrating system-specific conformational sampling. Similar FEL approaches have been used to complement conventional stability metrics in Keap1–peptide simulations [18,46].

Representative low-energy conformations were identified at 79.8 ns for apo Keap1 and at 63.7, 17.2, 75.9, 24.4, and 39.8 ns for the DHE-, HDHE-, DDHN-, EHED-, and QHDE-containing systems, respectively. Despite the different positions of their energy minima, the overall Keap1 fold remained well preserved, consistent with the RMSD, Rg, and SASA analyses. Differences in basin organization therefore reflect variation in conformational sampling among the systems rather than a direct quantitative ranking of peptide-binding strength.

The FEL patterns did not reproduce the Vina docking-score hierarchy, emphasizing that a favorable static docking pose does not necessarily correspond to a uniquely dominant trajectory-level conformational state. Docking and FEL analysis therefore provide complementary views of the systems: the former prioritizes static poses, whereas the latter describes conformational sampling during MD. Because neither directly establishes persistent peptide occupancy, peptide–Keap1 contacts were next evaluated through time-dependent hydrogen-bond counts and occupancy analysis.

**Figure 6:**
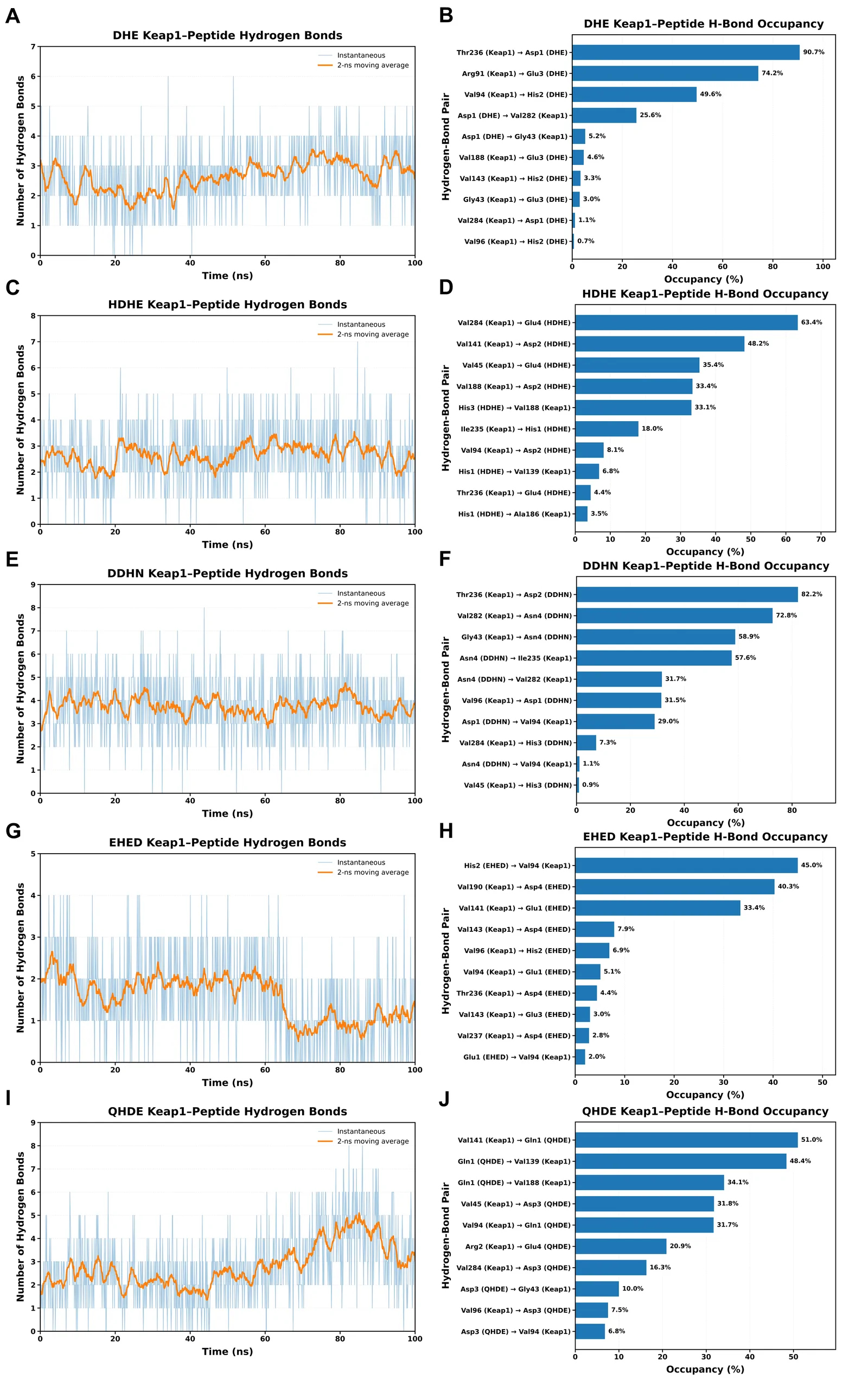
Hydrogen-bond dynamics and occupancy profiles of peptide–Keap1 complexes during molecular dynamics simulations. (**A**,**C**,**E**,**G**,**I**) Time-dependent numbers of peptide–Keap1 hydrogen bonds during the 100 ns simulations. (**B**,**D**,**F**,**H**,**J**) Occupancies of the major hydrogen bonds for DHE, DDHN, HDHE, EHED, and QHDE, respectively. Keap1 residues are numbered according to the MD topology; the corresponding original Keap1 residue number is obtained by adding 324 (e.g., Gly43 = Gly367 and Thr236 = Thr560). Peptide residues retain their original sequence positions.

Hydrogen-bond analysis revealed clear sequence-dependent differences in peptide–Keap1 interaction dynamics (Figure 6). DHE and HDHE generally maintained approximately 2–3 intermolecular hydrogen bonds during the 100 ns trajectories, whereas DDHN showed a more persistent network of approximately 3–4 bonds. EHED behaved differently, with its hydrogen-bond number declining markedly after approximately 60 ns. In contrast, QHDE developed additional hydrogen bonds during the latter half of the simulation and reached approximately 4–5 bonds around 80–90 ns. These time-dependent changes indicate that peptide–Keap1 contacts continuously rearranged, rather than remaining fixed in the initial docking geometry, consistent with previous studies in which persistent hydrogen-bond networks contributed to peptide association with Keap1 [18,44].

Occupancy analysis further resolved which contacts persisted most frequently during the trajectories (Figure 6B,D,F,H,J). DHE formed particularly persistent Keap1 Thr560–peptide Asp1 and Arg415–peptide Glu3 interactions, with occupancies of 90.7% and 74.2%, respectively. DDHN displayed several high-occupancy contacts, including Thr560–Asp2 (82.2%), Val606–Asn4 (72.8%), Gly367–Asn4 (58.9%), and peptide Asn4–Ile559 (57.6%), consistent with its relatively persistent hydrogen-bond network. HDHE was dominated by the Val608–Glu4 interaction (63.4%), whereas the two major EHED contacts reached only 45.0% and 40.3%. QHDE did not rely on a single dominant bond but maintained several moderate contacts, including Val465–Gln1 (51.0%) and peptide Gln1–Val463 (48.4%), in agreement with the broader network that emerged later in the simulation.

The dynamic contact patterns again differed from both the docking hierarchy and cellular efficacy. HDHE had the most favorable docking score, yet DHE and DDHN contained individual hydrogen bonds with higher occupancies, while QHDE broadened its hydrogen-bond network later in the trajectory. Most notably, EHED combined the least favorable docking score and declining late-stage hydrogen bonding with the greatest cytoprotective response in HepG2 cells. Thus, static docking, receptor-level MD metrics, and hydrogen-bond persistence each describe different aspects of peptide–Keap1 behavior, but none alone predicts cellular antioxidant performance. Across prediction, chemical assays, Keap1 interaction analyses, and cell viability, no single layer reproduced the others, supporting a multilevel interpretation in which these readouts are mechanistically informative but not interchangeable measures of antioxidant efficacy [18,44].

### 3.6. Effects of Selected Peptides on Keap1, Nrf2, and HO-1 Protein Expression

**Figure 7:**
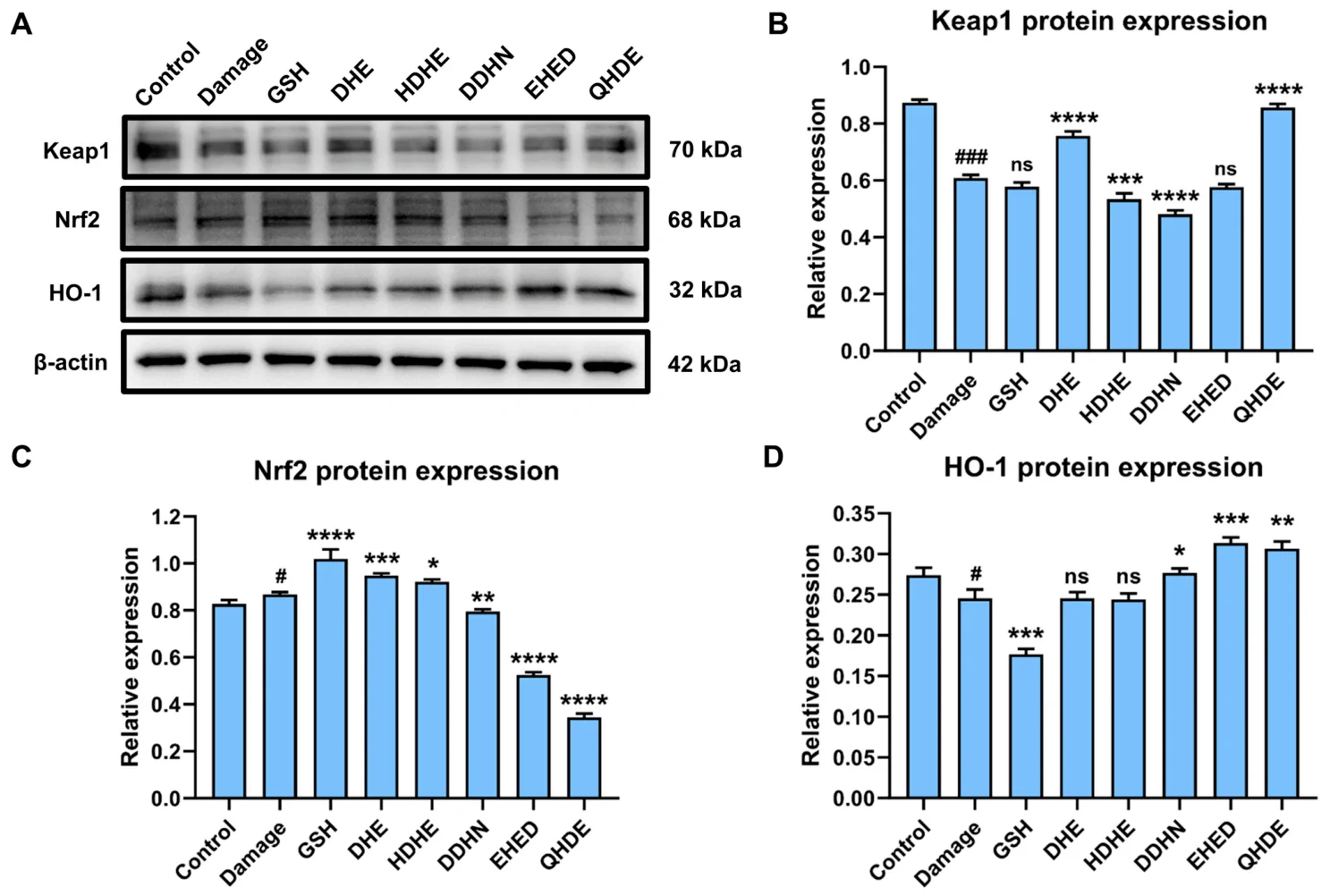
Effects of selected sea cucumber-derived peptides on Keap1, Nrf2, and HO-1 protein expression in D-galactose-treated HepG2 cells. (**A**) Representative Western blot bands of Keap1, Nrf2, HO-1, and β-actin in HepG2 cells. (**B**–**D**) Relative protein expression levels of Keap1, Nrf2, and HO-1, respectively, normalized to β-actin. Data are presented as mean ± SD from three independent biological replicates (*n* = 3). Statistical significance for the D-galactose damage group versus the control group is indicated by # (*p* ≤ 0.05) and ### (*p* ≤ 0.001). Statistical significance for each treatment group versus the D-galactose damage group is indicated by ns (*p* > 0.05), * (*p* ≤ 0.05), ** (*p* ≤ 0.01), *** (*p* ≤ 0.001), and **** (*p* ≤ 0.0001). GSH (2 mM) was included as a positive control, and the corresponding data are provided in the Supplementary Materials.

Western blot analysis showed sequence-dependent effects on Keap1, total Nrf2, and HO-1 expression (Figure 7). Relative to the control, D-galactose significantly decreased Keap1 and HO-1 while slightly increasing total Nrf2, so the three measured components did not change in parallel. DHE and QHDE significantly increased Keap1 relative to the damage group, whereas HDHE and DDHN decreased it and EHED caused no significant change. DHE and HDHE increased total Nrf2, while DDHN, EHED, and QHDE reduced it. In contrast, HO-1 was significantly increased by DDHN, EHED, and QHDE, with the greatest increases for EHED and QHDE; DHE and HDHE had no significant effect.

The protein-expression results sharpen the multilevel divergence observed throughout the study. EHED produced the greatest recovery of cell viability and the largest increase in HO-1 despite the least favorable docking score and relatively weak hydrogen-bond persistence. QHDE combined strong ABTS activity, favorable docking, a broader late-stage hydrogen-bond network, and increased HO-1. By contrast, HDHE led both DPPH scavenging and docking but did not significantly increase HO-1. Thus, predicted activity, chemical reactivity, Keap1 interaction characteristics, cellular protection, and downstream protein-expression responses were related but non-proportional dimensions of peptide antioxidant performance.

The canonical Keap1/Nrf2 pathway involves Nrf2 stabilization and nuclear translocation followed by transcriptional regulation of antioxidant-response genes such as HO-1, and antioxidant peptides can modulate Keap1/Nrf2-associated signaling [4,19,47]. Here, total Nrf2 did not consistently parallel HO-1, and total cellular Nrf2 does not necessarily represent the transcriptionally active nuclear pool. The data therefore demonstrate differential modulation of Keap1, total Nrf2, and HO-1 rather than canonical pathway activation. Increased HO-1 induced by DDHN, EHED, and QHDE may contribute to cytoprotection, but Nrf2 nuclear translocation and direct Keap1–Nrf2 interaction measurements would be required to establish pathway activation and causality.

## 4. Conclusions

This study established a proteome-scale in silico strategy and identified five previously uncharacterized *Apostichopus japonicus*-derived short peptides—DHE, HDHE, DDHN, EHED, and QHDE—with experimentally validated antioxidant activity. Virtual enzymatic hydrolysis, dual-model prediction, consensus ranking, toxicity screening, and structure-informed selection yielded five representative peptides—DHE, HDHE, DDHN, EHED, and QHDE—for experimental validation. All five showed measurable DPPH and ABTS radical-scavenging activity, good HepG2 cytocompatibility, and protection against D-galactose-induced loss of cell viability, with EHED providing the greatest recovery under the tested conditions.

Docking predicted sequence-dependent Keap1 interactions, while 100 ns MD simulations showed preserved global receptor stability and distinct time-dependent peptide–Keap1 hydrogen-bond patterns. These computational characteristics did not reproduce the chemical or cellular activity rankings. Western blotting likewise revealed sequence-dependent regulation of Keap1, total Nrf2, and HO-1, with DDHN, EHED, and QHDE significantly increasing HO-1. The results support contributions from both direct radical-scavenging activity and cellular antioxidant responses, but do not establish canonical Keap1/Nrf2/HO-1 pathway activation.

Several limitations remain. Intracellular ROS, antioxidant enzyme activities, Nrf2 nuclear translocation, and direct Keap1–Nrf2 interactions were not measured, limiting causal interpretation of cellular mechanism. Metal-chelating activity, peptide stability, and bioavailability also require direct evaluation. More broadly, the consistent discordance among prediction scores, radical-scavenging assays, Keap1 interaction metrics, and cellular outcomes demonstrates why antioxidant-peptide discovery should not rely on any single screening layer. Multilevel validation is therefore not only a confirmation step but a necessary strategy for distinguishing computationally prioritized sequences from peptides with meaningful cellular antioxidant performance.

## Figures and Tables

**Table 1:** In silico screening results, toxicity prediction, and activity scores of potential antioxidant peptides.

Sequence	Multi-AOP Probability	Pred5AOP Probability	Consensus Score	Toxicity
DHE	0.939	0.990	0.965	Non-toxic
DDHN	0.940	0.982	0.961	Non-toxic
HDHE	0.947	0.975	0.961	Non-toxic
HHED	0.945	0.975	0.960	Non-toxic
DHEN	0.942	0.970	0.956	Non-toxic
HHE	0.950	0.960	0.955	Non-toxic
DNHD	0.916	0.982	0.949	Non-toxic
EHED	0.916	0.978	0.947	Non-toxic
QHDE	0.951	0.942	0.947	Non-toxic
NHSD	0.946	0.945	0.945	Non-toxic

## Data Availability

The original contributions presented in this study are included in the article/Supplementary Material. Further inquiries can be directed to the corresponding authors.

## Supplementary Materials

The following supporting information can be downloaded at https://www.mdpi.com/article/10.3390/antiox15091188/s1, Figure S1. Antioxidant and cytoprotective effects of GSH. (A) DPPH and ABTS radical-scavenging activities of GSH at 2 mM. (B) Protective effect of 2 mM GSH against D-galactose-induced loss of HepG2 cell viability; Table S1. DPPH and ABTS radical-scavenging activities of the five selected peptides at different final concentrations; Table S2. Intrinsic absorbance of the selected peptides at 405 nm in the absence of ABTS•+; HPLC chromatograms and ESI-MS spectra of DHE, HDHE, DDHN, EHED, and QHDE.

## Abbreviations

The following abbreviations are used in this manuscript:

ABTS	2,2′-Azinobis(3-ethylbenzothiazoline-6-sulfonic acid)
DPPH	2,2-Diphenyl-1-picrylhydrazyl
GSH	Glutathione
Keap1	Kelch-like ECH-associated protein 1
Nrf2	Nuclear factor erythroid 2-related factor 2
HO-1	Heme oxygenase-1
MD	Molecular dynamics
RMSD	Root-mean-square deviation
RMSF	Root-mean-square fluctuation
PCA	Principal component analysis
FEL	Free-energy landscape
ROS	Reactive oxygen species
