# Supplementary material for: Proteome-Scale Discovery and Multilevel Validation of Antioxidant Short Peptides from Apostichopus japonicus: Keap1 Interactions and Cellular Antioxidant Responses
Source: Antioxidants (Basel). 2026 Sep 17;15(9):1188. doi: 10.3390/antiox15091188 (PMC13603116; doi:10.3390/antiox15091188)
Supplement: Supplementary file 1 [file antioxidants-15-01188-s001.zip › Supplementary_Tables_S1_S2_ThreeLine_SCI.pdf]

Table S1. DPPH and ABTS radical-scavenging activities of the five selected peptides at different final concentrations.

Peptide	Final concentration (mM)	DPPH radical-scavenging activity %, mean (95% CI)	ABTS radical-scavenging activity %, mean (95% CI)
DHE	0.125	8.20 (5.89–10.50)	18.21 (16.32–20.11)
	0.25	11.82 (7.34–16.30)	18.86 (17.07–20.64)
	0.5	14.79 (12.35–17.23)	20.42 (18.50–22.34)
	1	18.36 (15.87–20.85)	22.06 (19.66–24.46)
	2	21.55 (17.97–25.12)	23.27 (21.86–24.68)
HDHE	0.125	27.04 (24.42–29.65)	6.43 (3.99–8.86)
	0.25	29.13 (27.33–30.93)	8.08 (6.87–9.28)
	0.5	35.22 (30.63–39.81)	9.70 (8.21–11.18)
	1	36.69 (31.81–41.57)	11.43 (10.44–12.42)
	2	39.30 (36.07–42.53)	11.44 (10.23–12.66)
DDHN	0.125	16.54 (13.25–19.82)	15.54 (13.69–17.38)
	0.25	18.44 (16.47–20.40)	15.90 (15.09–16.71)
	0.5	21.52 (17.97–25.06)	18.43 (17.17–19.69)
	1	23.33 (19.88–26.77)	18.57 (17.06–20.09)
	2	25.32 (22.90–27.74)	20.13 (19.06–21.20)
EHED	0.125	13.49 (10.85–16.13)	15.10 (12.51–17.69)
	0.25	15.64 (12.23–19.05)	15.65 (13.68–17.62)
	0.5	18.95 (17.40–20.50)	19.22 (17.31–21.12)
	1	19.49 (18.00–20.98)	20.37 (18.50–22.25)
	2	23.53 (19.90–27.16)	23.68 (21.97–25.40)
QHDE	0.125	9.88 (6.44–13.33)	18.21 (16.46–19.96)
	0.25	13.32 (11.61–15.02)	18.07 (15.90–20.24)
	0.5	16.61 (13.18–20.04)	21.25 (18.40–24.10)
	1	20.28 (18.34–22.21)	27.85 (26.90–28.80)
	2	23.79 (19.78–27.80)	28.62 (26.61–30.64)

Note: Values are presented as mean (95% confidence interval), n = 4 per condition. Peptide concentrations are the final concentrations in the corresponding reaction mixtures. The 95% confidence interval was calculated as mean ± t0.975,3 × SD/√4.

Table S2. Intrinsic absorbance of the selected peptides at 405 nm in the absence of ABTS•+.

Peptide	A405 at final peptide concentration (mM)				
	0.125	0.25	0.5	1	2
DHE	0.0968 ± 0.0295	0.0703 ± 0.0045	0.0870 ± 0.0064	0.0753 ± 0.0030	0.0743 ± 0.0047
HDHE	0.0870 ± 0.0075	0.0803 ± 0.0144	0.0815 ± 0.0140	0.0943 ± 0.0029	0.0783 ± 0.0093
DDHN	0.0700 ± 0.0065	0.0650 ± 0.0034	0.0768 ± 0.0028	0.0733 ± 0.0041	0.0743 ± 0.0057
EHED	0.0778 ± 0.0081	0.0765 ± 0.0143	0.0768 ± 0.0102	0.0750 ± 0.0050	0.0690 ± 0.0047
QHDE	0.0763 ± 0.0163	0.0673 ± 0.0048	0.0813 ± 0.0046	0.0728 ± 0.0046	0.0700 ± 0.0024

Note: Values are mean ± SD from four replicate wells (n = 4). Each peptide was measured in the corresponding water/PBS vehicle without ABTS•+. The water/PBS vehicle blank measured in parallel was A405 = 0.079 ± 0.012.
