# Supplementary material for: Proteome-Scale Discovery and Multilevel Validation of Antioxidant Short Peptides from Apostichopus japonicus: Keap1 Interactions and Cellular Antioxidant Responses
Source: Antioxidants (Basel). 2026 Sep 17;15(9):1188. doi: 10.3390/antiox15091188 (PMC13603116; doi:10.3390/antiox15091188)
Supplement: Supplementary file 1 [file antioxidants-15-01188-s001.zip › Peptide_HPLC_QC/HPLC-DDHN.pdf]

生工® Sangon Biotech

Sample Information

Name	:	P42315-3
Sequence	:	DDHN
Modification	:	N/A
Lot.No	:	P42315-3-26062501
Pump A	:	0.1%trifluoroacetic in 100%water
Pump B	:	0.1%trifluoroacetic in 100%acetonitrile
Total Flow	:	1.0ml/min
Wavelength	:	214nm
Analytical column type	:	SHIMADZU shim-pack GIST C18(4.6*250MM*5UM)
Dissolution method	:	0.5mg sample dissolved to 0.5mL by 100%H2O
Acquisition Time	:	2026/09/08 09:29:30
Inj.Volume	:	30ul

Time	Module	Action	Value
0.01	Pumps	B.Conc	0
20.00	Pumps	B.Conc	20

Chromatogram

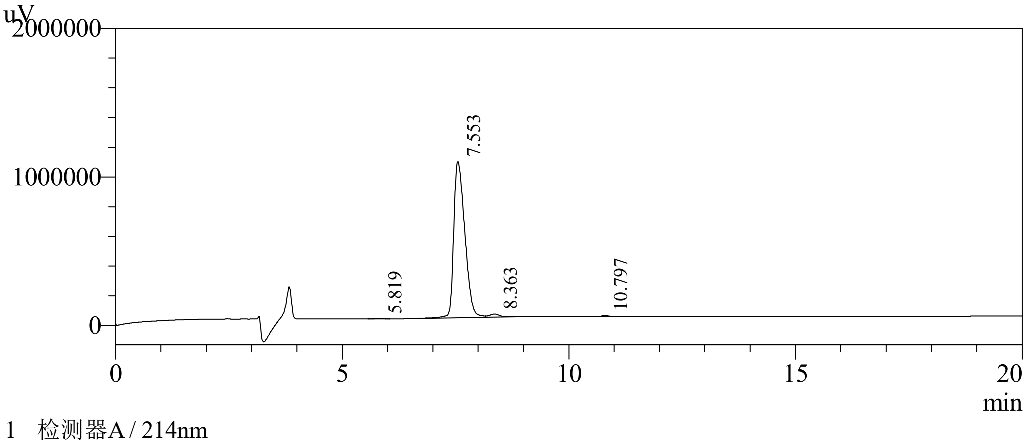

1 检测器A / 214nm

PeakTable

检测器A 214nm

Peak#	Ret. Time	Area	Height	Area %	Height %
1	5.819	24282	2249	0.132	0.208
2	7.553	17953703	1050820	97.308	96.991
3	8.363	385855	21481	2.091	1.983
4	10.797	86578	8868	0.469	0.819
总计		18450418	1083417	100.000	100.000
