# Supplementary material for: Proteome-Scale Discovery and Multilevel Validation of Antioxidant Short Peptides from Apostichopus japonicus: Keap1 Interactions and Cellular Antioxidant Responses
Source: Antioxidants (Basel). 2026 Sep 17;15(9):1188. doi: 10.3390/antiox15091188 (PMC13603116; doi:10.3390/antiox15091188)
Supplement: Supplementary file 1 [file antioxidants-15-01188-s001.zip › Peptide_HPLC_QC/HPLC-EHED.pdf]

生工® Sangon Biotech

Sample Information

Name : P42315-4
Sequence : EHED
Modification : N/A
Lot.No : P42315-4-26062501

Pump A : 0.1%trifluoroacetic in 100%water
Pump B : 0.1%trifluoroacetic in 100%acetonitrile
Total Flow : 1.0ml/min
Wavelength : 214nm
Analytical column type : SHIMADZU Inertsil ODS-SP(4.6*250MM*5UM)
Dissolution method : 0.5mg sample dissolved to 0.5mL by 100%H2O
Acquisition Time : 2026/09/08 09:30:00
Inj.Volume : 30ul

Time	Module	Action	Value
0.01	Pumps	B.Conc	0
20.00	Pumps	B.Conc	10

Chromatogram

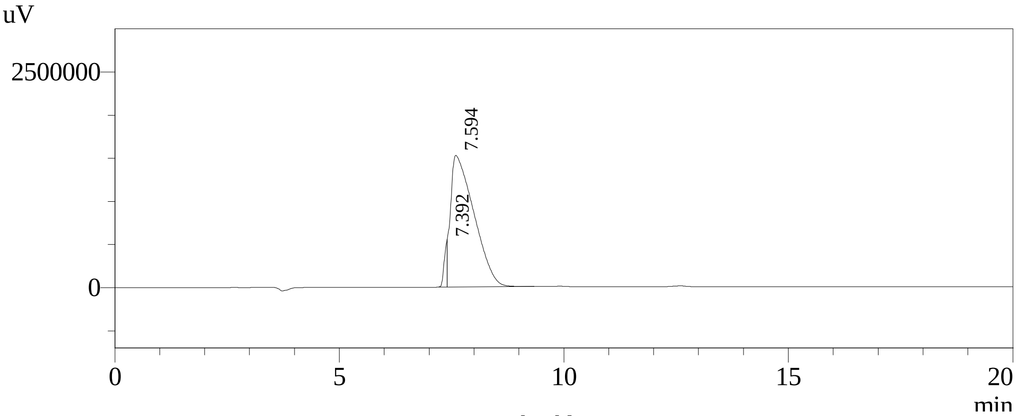

PeakTable

Detector A Ch1 214nm

Peak#	Ret. Time	Area	Height	Area %	Height %
1	7.392	2395728	526079	4.171	25.657
2	7.594	55037991	1524379	95.829	74.343
Total		57433718	2050458	100.000	100.000

生工生物工程（上海）股份有限公司

地址: 上海市松江区香闵路698号
电话/Tel: 400-821-0268
邮箱/Email: sales@sangon.com
Add: 698 Xiang Min Road SongJiang Shanghai China
传真/Fax: 86-21-37772170
网址/Web: www.sangon.com
