# Supplementary material for: Proteome-Scale Discovery and Multilevel Validation of Antioxidant Short Peptides from Apostichopus japonicus: Keap1 Interactions and Cellular Antioxidant Responses
Source: Antioxidants (Basel). 2026 Sep 17;15(9):1188. doi: 10.3390/antiox15091188 (PMC13603116; doi:10.3390/antiox15091188)
Supplement: Supplementary file 1 [file antioxidants-15-01188-s001.zip › Peptide_HPLC_QC/HPLC-HDHE.pdf]

生工® Sangon Biotech

## Sample Information

| | | |
|---|---|---|
| Name | : | P42315-2 |
| Sequence | : | HDHE |
| Modification | : | N/A |
| Lot.No | : | P42315-2-26062501 |
| Pump A | : | 0.1%trifluoroacetic in 100%water |
| Pump B | : | 0.1%trifluoroacetic in 100%acetonrtrile |
| Total Flow | : | 1.0ml/min |
| Wavelength | : | 214nm |
| Analytical column type | : | Waters xbridge C18 3.5um， 4.6mm*150mm Column |
| Dissolution method | : | 0.1mg sample dissolved to 0.5mL by 100%H2O |
| Acquisition Time | : | 2026/09/08 09:24:24 |
| Inj.Volume | : | 30ul |

| Time | Module | Action | Value |
|---|---|---|---|
| 0.01 | Pumps | B.Conc | 0 |
| 20.00 | Pumps | B.Conc | 5 |

## Chromatogram

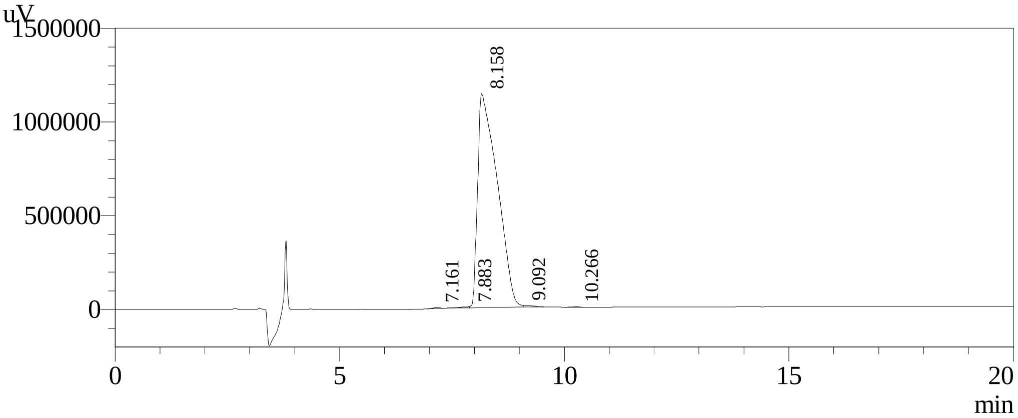


1 Det.A Ch1 / 214nm

## PeakTable

Detector A Ch1 214nm

| Peak# | Ret. Time | Area | Height | Area % | Height % |
|---|---|---|---|---|---|
| 1 | 7.161 | 55699 | 5675 | 0.160 | 0.487 |
| 2 | 7.883 | 81259 | 6315 | 0.233 | 0.543 |
| 3 | 8.158 | 34621900 | 1140819 | 99.189 | 98.002 |
| 4 | 9.092 | 118954 | 8404 | 0.341 | 0.722 |
| 5 | 10.266 | 27111 | 2863 | 0.078 | 0.246 |
| Total | | 34904923 | 1164076 | 100.000 | 100.000 |

**生工生物工程（上海）股份有限公司**

地址: 上海市松江区香闵路698号
电话/Tel: 400-821-0268
邮箱/Email: sales@sangon.com

Add: 698 Xiang Min Road SongJiang Shanghai China
传真/Fax: 86-21-37772170
网址/Web: www.sangon.com
