# Supplementary material for: Proteome-Scale Discovery and Multilevel Validation of Antioxidant Short Peptides from Apostichopus japonicus: Keap1 Interactions and Cellular Antioxidant Responses
Source: Antioxidants (Basel). 2026 Sep 17;15(9):1188. doi: 10.3390/antiox15091188 (PMC13603116; doi:10.3390/antiox15091188)
Supplement: Supplementary file 1 [file antioxidants-15-01188-s001.zip › Peptide_HPLC_QC/HPLC-QHDE.pdf]

生工® Sangon Biotech

Sample Information

Name	:	P42315-5
Sequence	:	QHDE
Modification	:	N/A
Lot.No	:	P42315-5-26062501
Pump A	:	0.1%trifluoroacetic in 100%water
Pump B	:	0.1%trifluoroacetic in 100%acetonrtrile
Total Flow	:	1.0ml/min
Wavelength	:	214nm
Analytical column type	:	Waters xbridge C18 3.5um， 4.6mm*150mm Column
Dissolution method	:	0.1mg sample dissolved to 0.5mL by 100%H2O
Acquisition Time	:	2026/09/08 09:25:08
Inj.Volume	:	30ul

Time	Module	Action	Value
0.01	Pumps	B.Conc	0
20.00	Pumps	B.Conc	5

Chromatogram

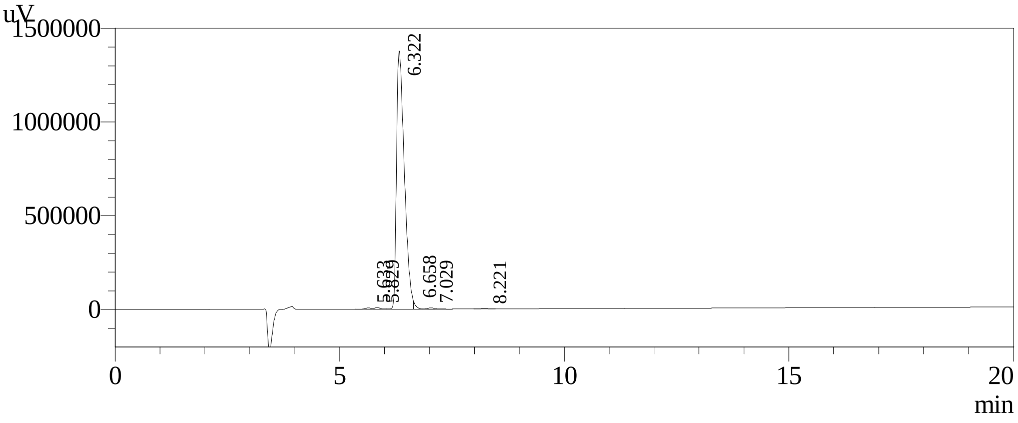

1 Det.A Ch1 / 214nm

PeakTable

Detector A Ch1 214nm

Peak#	Ret. Time	Area	Height	Area %	Height %
1	5.633	69755	7156	0.408	0.499
2	5.829	83779	8775	0.490	0.612
3	6.322	16736642	1376832	97.834	95.969
4	6.658	116805	33673	0.683	2.347
5	7.029	80746	6476	0.472	0.451
6	8.221	19432	1758	0.114	0.123
Total		17107159	1434670	100.000	100.000

生工生物工程（上海）股份有限公司

地址: 上海市松江区香闵路698号
电话/Tel: 400-821-0268
邮箱/Email: sales@sangon.com

Add: 698 Xiang Min Road SongJiang Shanghai China
传真/Fax: 86-21-37772170
网址/Web: www.sangon.com
