# Supplementary material for: Proteome-Scale Discovery and Multilevel Validation of Antioxidant Short Peptides from Apostichopus japonicus: Keap1 Interactions and Cellular Antioxidant Responses
Source: Antioxidants (Basel). 2026 Sep 17;15(9):1188. doi: 10.3390/antiox15091188 (PMC13603116; doi:10.3390/antiox15091188)
Supplement: Supplementary file 1 [file antioxidants-15-01188-s001.zip › Peptide_HPLC_QC/LCMS-DDHN.pdf]

生工® Sangon Biotech

MS Spectrum

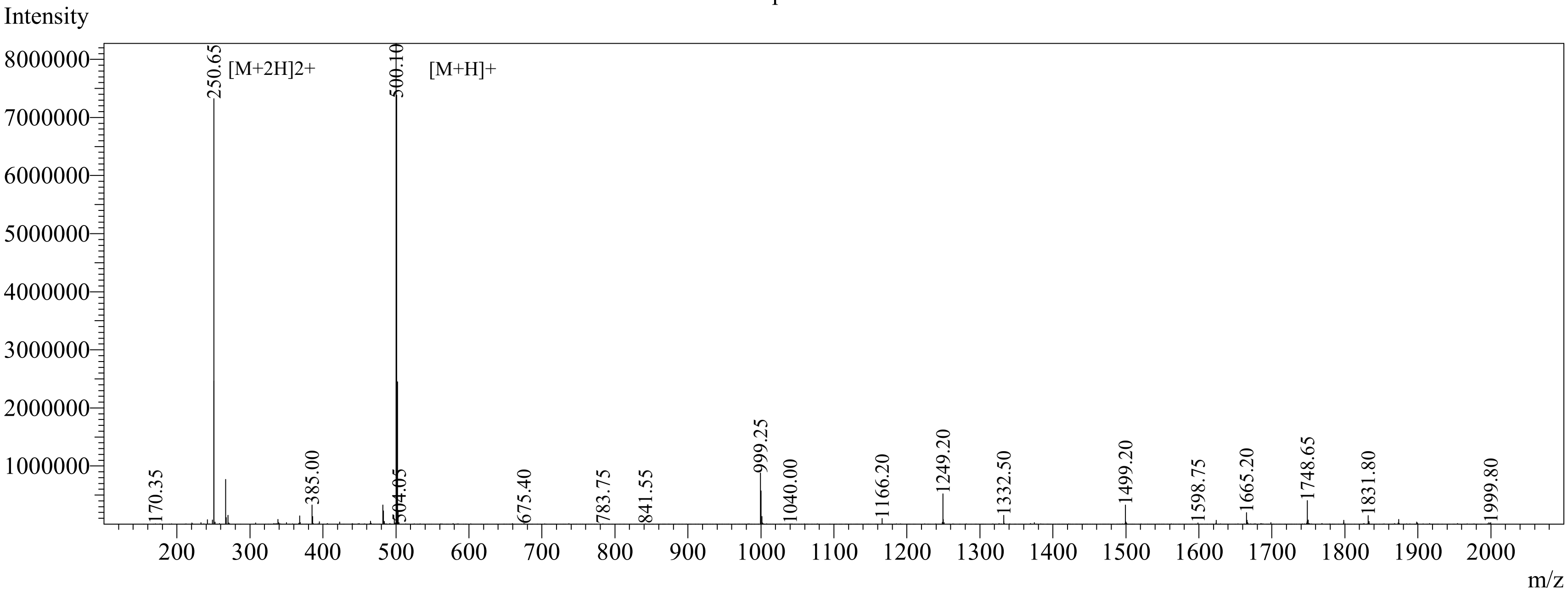

Sample Information		Interface	:ESI	Prerod Bias	:+4.5kv
Dissolution method	:0.1mg sample dissolved to 0.5mL by 50%ACN and 50%H2O	Nebulizing Gas Flow	:1.50L/min	Detector	:-0.2kv
		CDL Temp	:250℃	T.Flow	:0.2ml/min
Date Acquired	:2026/09/08 09:25:01	CDL Volt	:0v	B.conc	:0.001CH2O2 in 10%H2O/90%MeOH
Injection Volume	:1μl	Block Temp	:200		

Name	:P42315-3
Sequence	:DDHN
Modification	:N/A
Lot No.	:P42315-3-26062501
Theoretical	:499.423
Observed	:499.10

生工生物工程（上海）股份有限公司

地址：上海市松江区香闵路698号
Add: 698 Xiang Min Road Songjiang Shanghai China
电话/Tel: 400-821-0268
传真/Fax: 86-21-37772170
邮箱/Email: Sales@sangon.com
网址/Web: wwww.sangon.com
