# Supplementary material for: Proteome-Scale Discovery and Multilevel Validation of Antioxidant Short Peptides from Apostichopus japonicus: Keap1 Interactions and Cellular Antioxidant Responses
Source: Antioxidants (Basel). 2026 Sep 17;15(9):1188. doi: 10.3390/antiox15091188 (PMC13603116; doi:10.3390/antiox15091188)
Supplement: Supplementary file 1 [file antioxidants-15-01188-s001.zip › Peptide_HPLC_QC/LCMS-DHE.pdf]

生工® Sangon Biotech

MS Spectrum

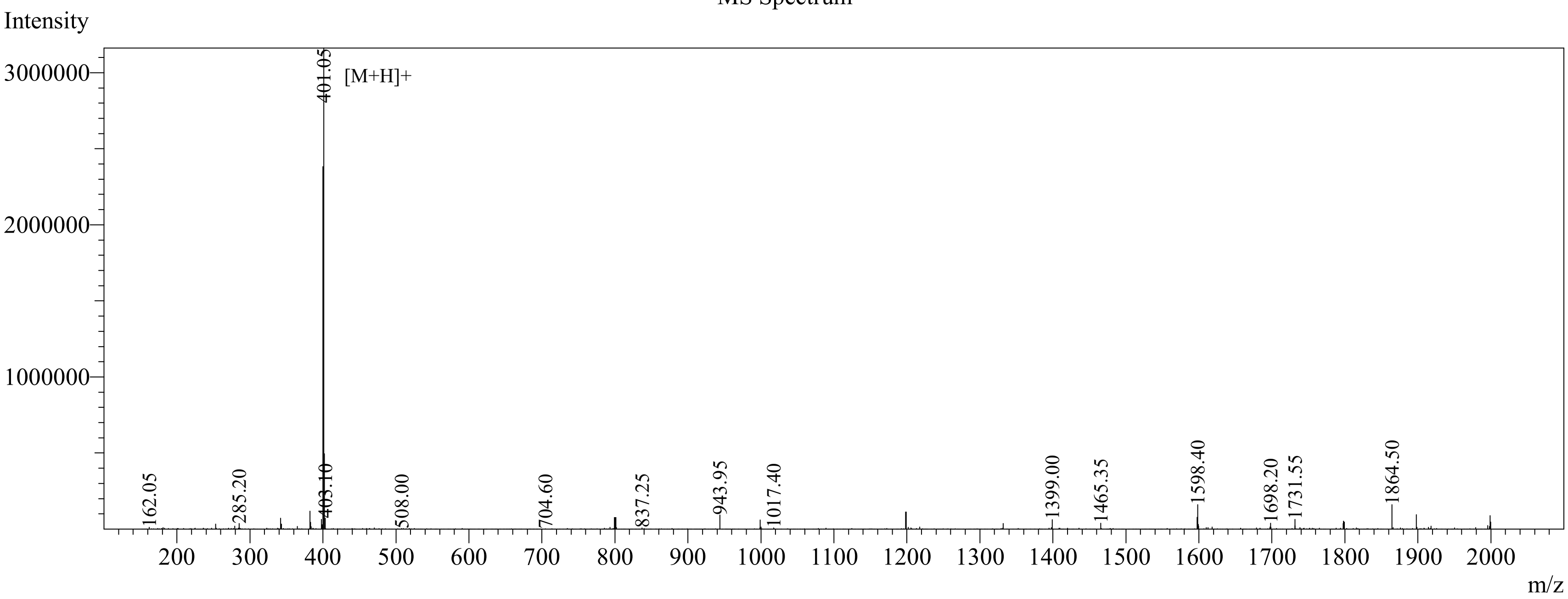

Sample Information
Dissolution method :0.1mg sample dissolved to 0.5mL by 50%ACN and 50%H2O
Date Acquired :2026/09/08 09:28:15
Injection Volume :1µl

Interface :ESI
Nebulizing Gas Flow :1.50L/min
CDL Temp :250℃
CDL Volt :0v
Block Temp :200

Prerod Bias :+4.5kv
Detector :-0.2kv
T.Flow :0.2ml/min
B.conc :0.001CH2O2 in 10%H2O/90%MeOH

Name :P42315-1
Sequence :DHE
Modification :N/A
Lot No. :P42315-1-26062501
Theoretical :399.346
Observed :400.05

生工生物工程（上海）股份有限公司

地址：上海市松江区香闵路698号
Add: 698 Xiang Min Road Songjiang Shanghai China
电话/Tel: 400-821-0268
传真/Fax: 86-21-37772170
邮箱/Email: Sales@sangon.com
网址/Web: wwww.sangon.com
